# Choice Architecture in Appalachian High Schools: Evaluating and Improving Cafeteria Environments

**DOI:** 10.3390/nu11010147

**Published:** 2019-01-11

**Authors:** Melissa D. Olfert, Rebecca L. Hagedorn, Emily N. Clegg, Shannon Ackerman, Cheryl Brown

**Affiliations:** 1 Department of Human Nutrition and Food, Division of Animal and Nutritional Sciences, Natural Resources and Design, Davis College of Agriculture, West Virginia University, Morgantown, WV 26506-6108, USA; rlhagedorn@mix.wvu.edu (R.L.H.); enclegg@mix.wvu.edu (E.N.C.); snackerman@mix.wvu.edu (S.A.); 2 Division of Resource Economics and Management, Natural Resources and Design, Davis College of Agriculture, West Virginia University, Morgantown, WV 26506-6108, USA; Cheryl.brown@mail.wvu.edu

**Keywords:** school lunch, choice architecture, behavioral economics, high school, Appalachia, adolescent health

## Abstract

School meals are a primary source of nutrition for many adolescents. Determining factors that influence the selection of various foods can provide insight on strategies to improve students’ cafeteria choices. This evaluation and observation was conducted at three Appalachian high schools to assess the cafeteria environment. The study developed and implemented an assessment tool created using principles of choice architecture and behavioral economics building on the work of the Cornell Center for Behavioral Economics in Child Nutrition Programs (BEN Center). The assessment tool scored eight components of the lunchroom—the exterior, hot serving area, cold serving area, salad bar, beverage area, payment station, dining area and grab-n-go, where a higher score equals healthier components offered. High school (HS) #1 earned 73/128 points (57%), HS #2 earned 69/128 (54%), and HS #3 earned 53/102 (52%). HS #3 did not have a grab-n-go option and the final score was out of 102. Video observation was used to collect data on lunchroom activity during mealtimes. Each school received reports that highlight the results and suggest improvements to raise their score. The scoring tool represents a novel way to assess the health of school lunches, provide insights on how to improve the healthfulness of students’ lunch choice, and improve overall nutrition status.

## 1. Introduction

The percentage of children with obesity in the United States has increased three-fold since the 1970s with one in five children ages 6–19 currently considered obese, and continuing to increase [1,2,3]. Specifically, obesity during adolescence, defined by the World Health Organization as 10–19 years of age [4], plays a detrimental impact on health and well-being, with adolescent obesity being related to increase prevalence of serious health complications that continue into adulthood [5,6]. Therefore, as the obesity epidemic continues, understanding factors influencing obesity prevalence among adolescents has been identified as a health priority. One of the primary factors that can impact adolescent weight status is diet. Specifically, low consumption of fruits and vegetables [7,8,9], sugar-sweetened beverage intake [10,11,12], and high energy intake [10,11,13,14] have been shown to be associated with increased weight status among adolescents. Therefore, there is a need to improve diet quality of adolescent youth to prevent obesity and associated health issues.

Adolescence is an ideal stage for nutrition intervention due to the developmental transitions that occur during this time. Adolescents have a need for independence [15] and, therefore, are often the main decision makers of their food intake, unlike that of younger children where parents are more heavily involved [16,17]. However, adolescence is also a period of behavior change, where social influences contribute to negative risk behaviors, and protective health behaviors are often overshadowed [18]. Thus, it is important to influence healthy dietary patterns in adolescences that could be sustained into adulthood as dietary behavior has been shown to sustain overtime [19]. One primary means of dietary intake for adolescents, and a potential target for intervention, is school lunch [18]. 

The National School Lunch Program (NSLP) provides approximately 31 million meals to students nationwide [20] and serves as a primary source of nutrition for many adolescents with approximately 50% of youths’ daily calories coming from school meals [21]. This makes school meals a prime target to understand and influence healthy food choices among adolescents. The United States Department of Agriculture (USDA) updates regulatory guidelines for the NSLP to align with the Dietary Guidelines for Americans [22]; however, children’s intake of fruit and vegetables is still below recommendations. The cafeteria environment often offers students have a variety of choices that can influence the nutritional quality of their meal and school policies to support healthy eating strategies are often lacking [23]. One strategy to help guide student’s towards healthy choices in the lunchroom is choice architecture. 

Choice architecture involves designing an environment in which selections are presented in a specific way so as to lead, or ‘nudge’, an individual to make a certain, desired choice [24,25]. Choice architecture is closely linked to the concept of behavioral economics, which takes all the factors that influence consumer’s choices (including price, appearance, state of mind, and habit) and attempts to create an environment that will encourage a better choice [26,27]. As champions in the choice architecture movement, research conducted by the Cornell Center for Behavioral Economics in Child Nutrition Programs (BEN Center) has shown that choice architecture and behavioral economics can impact food choice in school lunchrooms [28]. Since 2009, researchers at the BEN Center have studied how choice architecture and behavioral economics influence student’s choice in school lunchrooms. Through this research, the Smarter Lunchroom Movement (SLM) was founded, which offers low-cost options to alter school lunchrooms to encourage healthier choices by students. The SLM offers suggestions to improve school lunchrooms based on six core principles: convenience, portion sizes, availability, pricing strategies, taste expectations and selling techniques. Using these six principles, the SLM’s main objectives are to improve nutritional content of meals, maintain low cost, maintain participation in the NSLP, and encourage longer-term healthy decisions [29]. 

Determining factors that influence the selection of foods has the potential to provide insight on strategies to improve the healthfulness of students’ meal choices and implementation of SLM strategies has been well documented [30]. However, the use of choice architecture and behavioral economics strategies among Appalachian youth is less understood. Rural areas, specifically Appalachia, are of particular interest as rates of childhood obesity exceed the national average [31,32] and youth in rural regions are 25% more likely to be overweight or obese than their metropolitan counterparts [33]. This paper outlines the assessment of high-school cafeteria environments in Appalachia using the developed NudgeSAT assessment tool. The objective was to develop and implement a choice architecture assessment tool to evaluate Appalachian high school cafeterias and provide recommendations for improvement. 

## 2. Materials and Methods 

### 2.1. Study Design

This study was conducted in accordance with the Declaration of Helsinki and the protocol was deemed exempt by the Institutional Review Board at West Virginia University. This study includes the development and implementation of an observational assessment tool, NudgeSAT, and a video observation of cafeteria environments in three Appalachian high schools. A team of five researchers was assembled and trained to conduct the study. Training included an overview of the project, description of the assessment tool, and video camera usage. Researchers were instructed on the question types included in the assessment tool (Likert scale, yes/no, and multiple choice). Researchers arrived at the schools prior to lunch periods to disseminate the NudgeSAT assessment tool and video the cafeteria. Schools were provided with a recommendation for improvement based on results from the assessment and video observation. 

### 2.2. Site Identification and Recruitment

Local Appalachian high schools were identified as potential study sites to assess the cafeteria environment and the flow of students through the lunch line. The study was developed using components of community-based participatory research (CBPR). Stakeholders, including local Board of Education members (BOE), superintendents, county food service directors, cafeteria staff and school principals, were identified as being crucial for the success of the project. Permission to conduct the study was obtained from the BOE, and principals and cafeteria staff provided key information regarding school lunch operations. Five schools were initially identified for the study. However, one high school allowed students to leave the school campus during lunch. This school was the only one in the area to have this policy and was excluded based on this anomaly. Permission to conduct the study was not obtained from one of the identified sites, and thus three high schools were included in the study. Once sites were identified and permission had been obtained, a pre-observation was conducted at each site. Meetings were conducted with school principals and cafeteria staff. With their guidance, information was collected regarding the current cafeteria environment at each site. Pictures of each cafeteria were taken, outlines of cafeteria layout were drawn, and video camera placement was assessed. This information was crucial in the development of the written assessment tool, video assessment tool and post-site visit questionnaire. 

### 2.3. Assessment Tool Development

Authors undertook development of an assessment tool, NudgeSAT, to score high school cafeterias. The framework for the tool was adapted from research done by the BEN Center that uses principles of choice architecture and behavioral economics to improve cafeteria environments. This research identifies six main strategies to improve cafeterias: managing portion sizes, increasing convenience of healthy foods, improving visibility of healthy foods, enhancing taste expectations, utilizing suggestive selling and setting smart pricing strategies [29]. NudgeSAT took these six strategies and applied them to eight sections of a cafeteria: exterior, hot-serving area, cold-serving area, salad bar, beverage area, payment station, dining area and grab-n-go, as shown in Table 1. Questions were developed based on characteristics identified by the BEN Center as improving the cafeteria environment and formulated as yes/no, multiple choice or Likert scale questions. Point values were assigned to each section for a total of 128 possible points, with a higher score indicating more healthy components available. Each site could receive a total of 16 points in the exterior area components, 22 points in the hot serving area, 21 points from the cold serving area, 10 points in the salad bar area, 7 points for the beverage area, 5 points for the payment area, 20 points for the dining area, and 27 points for the grab and go component. The highest possible score was 128, although site #3 did not have a grab and go station causing their total score to be out of a possible 102 points. 

The questionnaire with assigned point values is available in Supplementary File 1. 

### 2.4. Video Observation 

Video observations were collected of the exterior area, serving area, dining area, and grab and go area for each lunch period. One researcher was assigned to each of these specific environments to record. The researchers that recorded the exterior and the serving environments stood on step stools to be able to observe the flow of the students through the environments. Each researcher recorded the same environment for every lunch period at each site. Video observations were collected during all lunch periods at sites #1 and #2, as there were three lunch periods, determined by where in the school the students were located. Site #3 had a smaller student population and only had one lunch period.

Following video collection, researchers completed a post-site visit questionnaire regarding the strengths, weaknesses and unique aspects found at each site. 

### 2.5. Data Analysis

The NudgeSAT was analyzed based on a points system. Yes or no questions were awarded 0 or 1 point, the point being awarded for conditions that indicated a positive aspect of the cafeteria’s choice architecture. The multiple-choice questions received 1 point for a specific answer chosen. The Likert scale questions received 2, 1, or 0 points. When assessing for noise, a no noise answer received 2 points, low to medium and medium received 1 point, and loud and very loud received 0 points. When assessing for lighting, no or low lighting received 0 points, low to medium and medium received 1 point, and bright and very bright received 2 points. The congestion question received 2 points for no congestion, 1 point for slight and neutral, and 0 points for congested and very congested. The food appearance question received 0 points for poor, 1 point for low to moderate and moderate, and 2 points for appealing and very appealing. The answer to each Likert scale question was based on the researchers’ opinion for that site. Each completed assessment was given an overall score and a percentage. A higher score represented a site that had more SLM components present. The highest score a site could receive was 128. 

The video clips were also analyzed separately for each high school with each of the four areas scored via three questions using a Likert scale system for 0, 1, or 2 points (more points indicating more health-promoting characteristics). Two registered dietitians who were familiar with SLM viewed segments of the videos that were representative of each high school’s lunchroom situation to get outside expert opinion on whether the high school cafeterias were meeting the criteria for a smarter lunchroom. Although the videos covered the full lunch period (videos were up to one hour in length) it was determined that a three-minute segment taken at the busiest time of each lunch period was sufficient to convey the factors that needed to be analyzed in order to give the cafeteria a smarter lunchroom score. Exterior, serving and grab and go (where present) areas were scored based on congestion, wait time and traffic pattern. Dining area was scored based on congestion, traffic pattern and amount of time students have to eat lunch. The highest possible video observation score was 24. The higher the score the smarter the lunchroom environment. The video assessment tool is shown in Table 2. 

The post-site visit answers were analyzed for common themes. NudgeSAT scores, video scores, and strengths and weaknesses for each site were compiled into individual site reports and these were presented to the principals of each high school.

## 3. Results

### 3.1. Site Demographics

Student numbers in each high school and school lunch sales differed among sites. Site #3 was a combined middle school and high school, however, only the high school lunch period was assessed as this study looked to implement the NudgeSAT tool in the high school population. The total number of lunches sold, the total number of students receiving free, reduced price, or full price lunch are shown in Table 3. For site #3, the school lunch participation could not be obtained for just the high school students and numbers represent middle and high school students combined. Students who did not participate in the NSL program either brought their own lunch or did not eat lunch. These numbers were obtained from the Food Service Director for the observational day for each site.

### 3.2. Written Assessment Tool

The written assessment tool was analyzed for each site and given a percentage score for each of the eight areas assessed and an overall score shown in Table 4. Areas of strength and weakness were able to be identified for each site. Site #1 received a total overall score of 57%, with the highest score for salad bar (90%) and lowest score for Grab and Go (44%). Site #2 also received its highest score for salad bar (80%) but had the lowest score for the payment station (40%) with an overall score of 54%. Site #3 did not have a grab and go section and had an overall total of 52%. Like sites #1 and 2, received its highest score for the salad bar (80%) but had the lowest score for the cold serving area. 

### 3.3. Video Recordings

Site #1 received a score of 5 for serving area, 4 for exterior, 3 for grab and go and 2 for dining area for a total score of 14/24 (58%). Site #2 received a 5 for grab and go, 4 for dining, and 2 for both serving and exterior for a total score of 13/24 (54%). Site #3 received a 5 for dining, and 2 for serving and exterior. Site #3 did not have a grab and go area, so their highest possible score was 18. Site #3 had a total score of 9/18 (50%). 

### 3.4. Post-Site Visit Questionnaire

Post-site visit questionnaires assessed strengths, weaknesses and unique aspects of each school lunchroom and were analyzed for common themes. Site #1 strengths were the availability of fresh fruits and vegetables in the salad bar and the efficiency of the payment station. Site #1 weaknesses were the inaccessibility of the salad bar and a lack of interactions between students and cafeteria staff. Site #2 was strong in lighting, amount of space in the cafeteria and pre-packaged fresh fruits and vegetables at the end of the serving line. Site #2’s largest weakness was a large plastic shield across the salad bar making it difficult for students to access. Site #3 was strong in cleanliness and staff friendliness. Weaknesses of site #3 were congestion and limited space in the serving area.

### 3.5. Recommendation Reports

**Figure 1:**
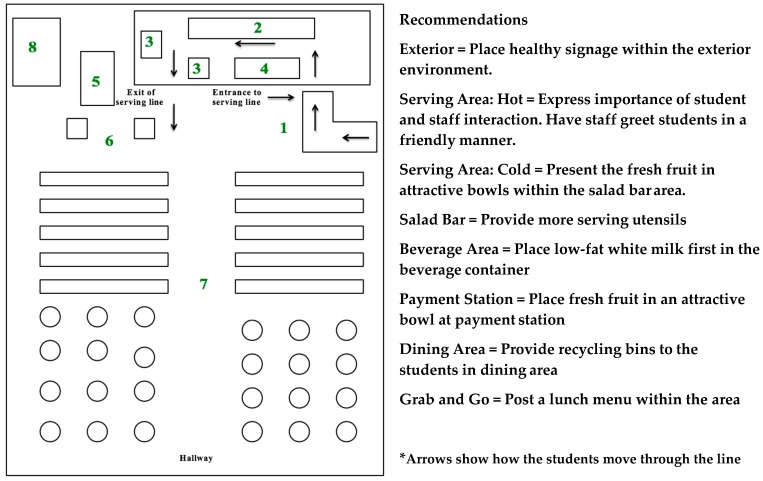
Recommendation report to improve NudgeSAT score at Site 2.

Based on the combined results of the written assessment tool, video observations and post-visit questionnaires, recommendation reports were created for administrators at each high school. Additionally, a monetary donation was provided to each school to implement recommended changes in order to improve their cafeteria environment and encourage healthier food choices by high school students. A sample recommendation is shown in Figure 1. 

## 4. Discussion

This study provided insight on three Appalachian cafeterias and results showed all three sites to be lacking a substantial number of choice architecture components. Sites scored best on salad bar components, but each site had a different area that it struggled the most in-site #1 in grab and go (44%), site #2 in payment station (40%) and site #3 cold serving area (33%) and have different focuses for improvement. Video observation results mirrored the findings of the NudgeSAT with site #1 scoring 57% on the NudgeSAT and 58% in the video observations, site #2 scoring 54% on both the NudgeSAT and in video recordings, and site #3 scoring 52% on NudgeSAT and 50% on video. While overall scores were similar for NudgeSAT and video observations, there were some differences in the individual components of each. For site #1, in the NudgeSAT the serving areas received a lower score, but in video observation, received a higher score. This was also seen in site #2’s grab and go, where in NudgeSAT it received a low score but was a stronger component in video observation. These differences can potentially be explained by differences in what each tool was capturing, where the NudgeSAT focuses on components present in each area of the lunchroom and the video observation focuses on the dynamics of the cafeteria environment.

The use of choice architecture interventions among youth is growing in many developed countries, yet studies in the high school setting are limited [25]. One study in secondary schools in the United Kingdom found that students at choice architecture intervention schools were 2.5 times as likely to select healthier food options [34]. This highlights the potential impact of choice architecture on adolescent dietary behaviors, although more studies are needed. 

Understanding the components of the cafeteria environment can help researchers to identify areas to implement and improve choice architecture [35]. Currently, the literature suggests modifications to the cafeteria environment can improve food choice behaviors and should be considered in interventions [36]. This includes small changes, such as healthy and unhealthy food placement, verbal prompting, signage location and food labeling, and use of nudges, as ways to influence a healthy food choice [24,37,38,39,40]. Meiselman et al. highlight that simply rearranging the location of healthy and unhealthy selections (i.e., moving potato chips to a more distal location) can increase the purchase of healthy options by making the healthy choice the easy choice [41]. This easy choice is chosen even when unhealthy options remain, as the convenient choice seems to be the first choice [38,42]. Furthermore, Thorndike et al. encourage the use of labeling to help students easily select the healthy choice which has been shown to increase the sales of healthy items [24]. These changes can be made to various areas of the lunchroom setting, making it important to understand which components of the cafeteria environment need change. 

The NudgeSAT tool proposed here can aid schools in understanding which areas of their cafeteria could be improved upon and implement change to help students make the healthy, easy choice. The literature on choice architecture is growing and if used in combination with our assessment, interventions could possibly be planned to identify and design interventions in order to improve school lunch decisions in Appalachia. It is the hope of researchers to identify strengths in NudgeSAT to design an effective intervention to help combat the rising rates of obesity in the Appalachian region. 

At the time of this study, there was no validated tool available to assess the school lunchroom environment and the development of the assessment tool for this study represented the first of its kind. Since the conclusion of this study, the SLM has created their own assessment tool, the Smarter Lunchrooms Self-Assessment Scorecard [43]. The SLM tool is 60 questions regarding the cafeteria environment. The SLM Self-Assessment Scorecard questions are all in a checklist format, where items that are present are checked off. A score is given based on the number of items checked and the lunchroom is designated as gold, silver or bronze. The NudgeSAT tool includes several factors not included in the SLM scorecard. These include level of congestion in the lunch line, presence of pleasant or unpleasant orders in the lunchroom, noise level and lighting. These components are important to consider as they have been shown to influence food intake and food choice [44]. The addition of these factors creates a more comprehensive picture of the cafeteria environment. Future research could compare the usability of these tools and make improvements for one robust tool to improve cafeteria environments.

This study is not without limitations. First, this study was conducted at three high schools in the same county in Appalachia and, therefore, calls for testing of the tool in other areas in Appalachia. Additionally, the dining environments of school cafeterias vary between elementary, middle, and high school, which limits these results to a high school population only. Additional testing in elementary and middle school populations in Appalachia is warranted. In addition, each site was only observed for one day and additional observation would improve the reliability and validity of the assessment tool. This calls for future research to facilitate validation of this tool. While the assessment tool captured many aspects of the school lunchroom, it may not be specific enough to completely capture what was observed at each site. All three sites had the same score for dining on the assessment tool, however each site had very different dining areas with their own strengths and weaknesses, which was not captured in the scores given by the assessment tool. Given the potential of this tool to assess cafeteria environments and provide recommendations for improvements, it may be warranted to document any policy, system and environmental (PSE) changes that occur in the high schools as a result of these recommendation reports.

## 5. Conclusions

This study provides an important first effort to understand the role of choice architecture in food choice in Appalachian high schools. Limited work has been done to understand the use of SLM strategies in rural high schools [45], and the creation of NudgeSAT represents the first study to seek to understand choice architecture in Appalachian high schools. The study provides an important base of information to inform future research. Future directions for this work should look to understand differences between Appalachian and non-Appalachian high school cafeteria environments and interventions should address these differences. By assessing lunchrooms with NudgeSAT, researchers can identify specific strengths and weaknesses to that cafeteria and, in turn, design low-cost interventions to improve the cafeteria environment. This study provided recommendation reports to empower high schools to make low-cost changes to improve their cafeteria environment using choice architecture principles. 

## Figures and Tables

**Table 1:** NudgeSAT evaluation components and total points for each area of the cafeteria environment.

	Exterior	Serving Area: Hot	Serving Area: Cold	Salad Bar	Beverage Area	Payment Method	Dining Area	Grab and Go
**Total Points Possible**	16	22	21	10	7	5	20	27
**Components evaluated**	Congestion	Staff friendliness	Staff friendliness	Traffic pattern	Order of milk type	Efficiency	Consumption	Availability of FFV
	Lunch menu	Tray color	Food appearance	Accessibility	50% white milk	Congestion	Availability of trash cans	Traffic pattern
	Wait time	Food appearance	Availability of FFV	Food appearance	Traffic-pattern	Traffic-pattern	Lighting	Beverage area
	Odor	Availability of FFV	Noise level	Cleanliness	Congestion	Availability of FFV	Noise Level	Efficiency of payment
	Lighting	Noise level	Congestion	Availability of FFV			Time	
	Noise level	Congestion	Traffic pattern				Overall tray waste	
	Traffic pattern	Traffic pattern						

Note: Fresh fruit and vegetable is abbreviated FFV.

**Table 2:** Video assessment tool evaluation content for each area of the cafeteria environment.

	Exterior	Serving	Dining	Grab and Go
**Congestion**	Very Congested (0) Congested (0) Neutral (1) Slightly Congested (2) No Congestion (2)	Very Congested (0) Congested (0) Neutral (1) Slightly Congested (2) No Congestion (2)	Very Congested (0) Congested (0) Neutral (1) Slightly Congested (2) No Congestion (2)	Very Congested (0) Congested (0) Neutral (1) Slightly Congested (2) No Congestion (2)
**Wait time**	>3 min (0) >2 min (0) >1 min (1) >30 s (2) No Wait Time (2)	>3 min (0) >2 min (0) >1 min (1) >30 s (2) No Wait Time (2)	-	>3 min (0) >2 min (0) >1 min (1) >30 s (2) No Wait Time (2)
**Traffic pattern**	Not Efficient (0) Slightly Efficient (0) Neutral (1) Efficient (2) Very Efficient (2)	Not Efficient (0) Slightly Efficient (0) Neutral (1) Efficient (2) Very Efficient (2)	Not Efficient (0) Slightly Efficient (0) Neutral (1) Efficient (2) Very Efficient (2)	Not Efficient (0) Slightly Efficient (0) Neutral (1) Efficient (2) Very Efficient (2)
**Amount of time to eat lunch**	-	-	<16 min (0) 17 min (0) 18 min (1) 19 min (2) 20 min (2)	-

**Table 3:** School-level demographics of the three participating sites.

Site	# Students in the High School	# of Students Participating in NSLP	Free Lunch Participation	Reduced Lunch Participation	Paid Full Price for Lunch
**1**	1656	879 (53%)	240 (27%)	43 (5%)	596 (68%)
**2**	1251	746 (60%)	184 (25%)	53 (7%)	509 (68%)
**3**	452	347 (77%) *	112 (32%) *	27 (8%) *	208 (60%) *

Note: The percentage for the number of students who participate in the National School Lunch Program is out of the number of students in the high school. The percentages for Free Lunch Participation, Reduced Price Lunch Participation, and Paid Full Price for Lunch are out of the number of students who participate in the National School Lunch Program. Information provided by School Food Service Director. * includes both middle and high school students. # indications the number of students.

**Table 4:** NudgeSAT results for each area of the cafeteria environment from all sites.

	Exterior (16)	Serving Area: Hot (22)	Serving Area: Cold (21)	Salad Bar (10)	Beverage Area (7)	Payment Station (5)	Dining Area (20)	Grab and Go (27)	Total
**Site 1**	9 (56%)	10 (45%)	10 (47%)	9 (90%)	6 (86%)	4 (80%)	13 (65%)	12 (44%)	73/128 (57%)
**Site 2**	8 (50%)	9 (41%)	11 (52%)	8 (80%)	4 (57%)	2 (40%)	13 (65%)	14 (42%)	69/128 (54%)
**Site 3**	11 (69%)	8 (36%)	7 (33%)	8 (80%)	3 (43%)	3 (60%)	13 (65%)	N/A	53/102 (52%)

Note: Numbers in parentheses are the maximum amount of points that could be awarded for that component. Percentages in parentheses are the percentages for that specific component.

## Supplementary Materials

The following are available online at http://www.mdpi.com/2072-6643/11/1/147/s1, Supplementary File 1: NudgeSAT Assessment Tool.
