# Supplementary material for: Choice Architecture in Appalachian High Schools: Evaluating and Improving Cafeteria Environments
Source: Nutrients. 2019 Jan 11;11(1):147. doi: 10.3390/nu11010147 (PMC6356853; doi:10.3390/nu11010147)
Supplement: Supplementary file 1 [file nutrients-11-00147-s001.pdf]

Supplementary File 1: NudgeSAT Assessment Tool

What high school did you observe?

Exterior Area (16 Points):

Are there "healthy" posters or signage within the area? (healthy defined as nutritional or promoting a healthy lifestyle)

- ❍ Yes (1)
- ❍ No (0)

Is there a lunch menu posted within the area?

- ❍ Yes (1)
- ❍ No (0)

Is the lunch menu neat and clear to read?

- ❍ Yes (1)
- ❍ No (0)

Is the area where the utensils are located, clean and free from clutter?

- ❍ Yes (1)
- ❍ No (0)

Are there food placement directions for the tray to inform students where to place each type of food and milk?

- ❍ Yes (1)
- ❍ No (0)

Are there any "verbal prompts" being provided to the students while they stand in line? (A verbal prompt is any statement about the food that would affect the student’s choice)

- ❍ Yes (1)
- ❍ No (0)

Does the line have a clear traffic pattern?

- ❍ Yes (1)
- ❍ No (0)

How would you rate the level of congestion within the line?

	No Congestion (2)	Slightly Congested (1)	Neutral (1)	Congested (0)	Very Congested (0)
Level of Congestion (1)	❍	❍	❍	❍	❍

How would you rate the noise level within the area?

	No/Low (2)	Low to Medium (1)	Medium (1)	Loud (0)	Very Loud (0)
Noise Level (1)	❍	❍	❍	❍	❍

Did you detect any negative odor?

- ❍ Yes (0)
- ❍ No (1)

How would you rate the level of the food preparation smell?

	No Smell (2)	Slight Smell (1)	Neutral (1)	Favorable Smell (0)	Very Favorable Smell (0)
Level of Smell (1)	❍	❍	❍	❍	❍

How would you rate the level of lighting within the area?

	No/Low (2)	Low/Medium (1)	Medium (1)	Bright (0)	Very Bright (0)
Lighting Level (1)	❍	❍	❍	❍	❍

Serving Area Hot (22 Points):

What is the color of the tray?

- ❍ White (0)
- ❍ Black (0)
- ❍ Yellow (0)
- ❍ Red (1)
- ❍ Other (0)

Food appearance?

	Poor (0)	Low to Moderate (1)	Moderate (1)	Appealing (2)	Very Appealing (2)
Food Appearance Rating (1)	❍	❍	❍	❍	❍

Is fresh fruit available in more than one place?

- Yes (1)
- No (0)

Where is the fresh fruit displayed? (Check all that apply)

- At the beginning of the line (1)
- At the end of the line (0)
- In another area of the line (0)

How is the fresh fruit displayed?

- Attractive bowls (1)
- Stainless steel containers (0)
- Clear plastic bins (0)
- Other (0)

Are fresh vegetables available in more than one place?

- Yes (1)
- No (0)

Where are the fresh vegetables displayed within in the line?

- At the beginning of the line (1)
- At the end of the line (0)
- In another area of the line (0)

How are the fresh vegetables displayed?

- In attractive bowls (1)
- In stainless steel containers (0)
- In clear plastic bins (0)
- Other (0)

Are the fresh fruit and vegetables easy to access?

- Yes (1)
- No (0)

Are the students greeted by the school staff as they enter the serving area?

- Yes (1)
- No (0)

Is the staff cheerful?

- Yes (1)
- No (0)

Are the students prompted by the staff to take fresh fruit?

- Yes (1)
- No (0)

Are the students prompted by the staff to take fresh vegetables?

- ❍ Yes (1)
- ❍ No (0)

Is the area where the hot food is being served kept clean and free from spills?

- ❍ Yes (1)
- ❍ No (0)

Does the line have a clear traffic pattern?

- ❍ Yes (1)
- ❍ No (0)

How would you rate the level of congestion within the line?

	No Congestion (2)	Slightly Congested (1)	Neutral (1)	Congested (0)	Very Congested (0)
Level of Congestion (1)	❍	❍	❍	❍	❍

How would you rate the noise level within the area?

	No/Low (2)	Low to Medium (1)	Medium (1)	Loud (0)	Very Loud (0)
Noise Level (1)	❍	❍	❍	❍	❍

How would you rate the level of lighting within the area?

	No/Low (0)	Low to Medium (1)	Medium (1)	Bright (2)	Very Bright (2)
Level of Lighting (1)	❍	❍	❍	❍	❍

<u>Serving Area Cold (21 Points):</u>

Food appearance?

	Poor (0)	Low to Moderate (1)	Moderate (1)	Appealing (2)	Very Appealing (2)
Food Appearance Rating (1)	❍	❍	❍	❍	❍

Is fresh fruit available in more than one place?

- Yes (1)
- No (0)

Where is the fresh fruit displayed within the line?

- At the beginning of the line (1)
- At the end of the line (0)
- In another area of the line (0)

How is the fresh fruit displayed?

- In attractive bowls (1)
- In stainless steel containers (0)
- In plastic bins (0)
- Other (0)

Are fresh vegetables available in more than one place?

- Yes (1)
- No (0)

Where are the fresh vegetables displayed within the line?

- At the beginning of the line (1)
- At the end of the line (0)
- In another area of the line (0)

How are the fresh vegetables displayed?

- In attractive bowls (1)
- In stainless steel containers (0)
- In clear plastic bins (0)
- Other (0)

Are fresh fruit and vegetables easy to access?

- Yes (1)
- No (0)

Are the students greeted by staff as they enter the serving area?

- Yes (1)
- No (0)

Is the staff cheerful?

- Yes (1)
- No (0)

Are the students prompted by the staff to take fresh fruit?

- Yes (1)
- No (0)

Are the students prompted by the staff to take fresh vegetables?

- ❍ Yes (1)
- ❍ No (0)

Is the area where the cold food is being served kept clean and free from spills?

- ❍ Yes (1)
- ❍ No (0)

Does the line have a clear traffic pattern?

- ❍ Yes (1)
- ❍ No (0)

How would you rate the level of congestion within the line?

	No Congestion (2)	Slightly Congested (1)	Neutral (1)	Congested (0)	Very Congested (0)
Level of Congestion (1)	❍	❍	❍	❍	❍

How would you rate the noise level within the area?

	No/Low (2)	Low to Medium (1)	Medium (1)	Loud (0)	Very Loud (0)
Noise Level (1)	❍	❍	❍	❍	❍

How would you rate the level of lighting within the area?

	No/Low (0)	Low to Medium (1)	Medium (1)	Bright (2)	Very Bright (2)
Level of Lighting (1)	❍	❍	❍	❍	❍

<u>Salad Bar Area (10 Points):</u>

Is there a salad bar?

- ❍ Yes (1)
- ❍ No (0)

Is the salad bar easily accessible?

- ❍ Yes (1)
- ❍ No (0)

Are the self-serve tongs/scoops easy to use?

- ❍ Yes (1)
- ❍ No (0)

Is the salad bar kept clean and free from spills?

- ❍ Yes (1)
- ❍ No (0)

Are there enough serving tongs/scoops to use?

- ❍ Yes (1)
- ❍ No (0)

Is there a clear traffic pattern to the salad bar?

- ❍ Yes (1)
- ❍ No (0)

How would you rate the level of congestion within the area?

	No Congestion (2)	Slightly Congested (1)	Neutral (1)	Congested (0)	Very Congested (0)
Level of Congestion (1)	❍	❍	❍	❍	❍

How would you rate the level of lighting within the area?

	No/Low (0)	Low to Medium (1)	Medium (1)	Bright (2)	Very Bright (2)
Level of Lighting (1)	❍	❍	❍	❍	❍

Beverage Area (7 Points):

Are there posters or signage promoting white milk consumption?

- ❍ Yes (1)
- ❍ No (0)

Is the beverage container filled with at least 50% white milk?

- ❍ Yes (1)
- ❍ No (0)

Is the white milk easily accessible?

- ❍ Yes (1)
- ❍ No (0)

Is there a clear traffic pattern to the milk?

- ❍ Yes (1)
- ❍ No (0)

As the students approach the beverage container, is the white milk offered first?

- ❍ Yes (1)
- ❍ No (0)

How would you rate the level of congestion within the area?

	No Congestion (2)	Slightly Congested (1)	Neutral (1)	Congested (0)	Very Congested (0)
Level of Congestion (1)	❍	❍	❍	❍	❍

Payment Area (5 points):

Are there fresh fruit or vegetables offered at the payment station?

- ❍ Yes (1)
- ❍ No (0)

Is there a clear traffic pattern to the payment station?

- ❍ Yes (1)
- ❍ No (0)

How would you rate the level of congestion within the area?

	No Congestion (2)	Slightly Congested (1)	Neutral (1)	Congested (0)	Very Congested (0)
Level of Congestion (1)	❍	❍	❍	❍	❍

Is the payment method efficient?

- ❍ Yes (1)
- ❍ No (0)

Dining Area (20 points):

Are there "healthy" posters or signage within the area? (healthy defined as nutritional or promoting a healthy lifestyle)

- ❍ Yes (1)
- ❍ No (0)

Is tomorrow's lunch menu posted?

- ❍ Yes (1)
- ❍ No (0)

Is the menu clear and neat?

- ❍ Yes (1)
- ❍ No (0)

Is there a clear traffic pattern into the dining area?

- ❍ Yes (1)
- ❍ No (0)

How would you rate the level of congestion?

	No Congestion (2)	Slightly Congested (1)	Neutral (1)	Congested (0)	Very Congested (0)
Level of Congestion (1)	❍	❍	❍	❍	❍

How would you rate the level of lighting within the area?

	No/Low (0)	Low to Medium (1)	Medium (1)	Bright (2)	Very Bright (2)
Lighting Level (1)	❍	❍	❍	❍	❍

How would you rate the noise level within the area?

	No/Low (2)	Low to Medium (1)	Medium (1)	Loud (0)	Very Loud (0)
Noise Level (1)	❍	❍	❍	❍	❍

Do you detect any negative smell?

- ❍ Yes (0)
- ❍ No (1)

How would you rate the level of food smell within the area?

	No Smell (2)	Slight Smell (1)	Neutral (1)	Favorable Smell (0)	Very Favorable Smell (0)
Level of Smell (1)	❍	❍	❍	❍	❍

Is the garbage area kept clean?

- ❍ Yes (1)
- ❍ No (0)

Did you notice any food waste?

- ❍ Yes (0)
- ❍ No (1)

If yes, then what percentage of food was wasted?

- ❍ 0-25% (2)
- ❍ 26%-50% (0)
- ❍ 51%-75% (0)
- ❍ 76%-100% (0)

Is there recycling available?

- ❍ Yes (1)
- ❍ No (0)

Is there teaching staff present?

- ❍ Yes (1)
- ❍ No (0)

Is there administration staff present?

- ❍ Yes (1)
- ❍ No (0)

Do the students have an adequate amount of time to eat their lunch? (i.e. 20 minutes)

- ❍ Yes (1)
- ❍ No (0)

Grab n Go Area (27 Points):

Is there a grab and go option?

- ❍ Yes (1)
- ❍ No (0)

Is there a lunch menu posted within the area?

- ❍ Yes (1)
- ❍ No (0)

Is the menu clear and neat?

- ❍ Yes (1)
- ❍ No (0)

Food Appearance?

	Poor (0)	Low to Moderate (1)	Moderate (1)	Appealing (2)	Very Appealing (2)
Click to write Statement 1 (1)	❍	❍	❍	❍	❍

Is fresh fruit available in more than one place?

- ❍ Yes (1)
- ❍ No (0)

Where is fresh fruit displayed?

- ❍ At the beginning of the line (1)
- ❍ At the end of the line (0)
- ❍ In another area of the line (0)

How is the fresh fruit displayed?

- ❍ In attractive bowls (1)
- ❍ In stainless steel bins (0)
- ❍ In clear plastic containers (0)
- ❍ Other (0)

Are fresh vegetables available in more than one place?

- ❍ Yes (1)
- ❍ No (0)

Where are the fresh vegetables displayed?

- ❍ At the beginning of the line (1)
- ❍ At the end of the line (0)
- ❍ In another area of the line (0)

How are the fresh vegetables displayed?

- ❍ In attractive bowls (1)
- ❍ In stainless steel containers (0)
- ❍ In clear plastic bins (0)
- ❍ Other (0)

Are fresh fruit and vegetables easy to access?

- ❍ Yes (1)
- ❍ No (0)

Is the beverage container filled with at least 50% white milk?

- ❍ Yes (1)
- ❍ No (0)

As the students approach the beverage container, is the white milk offered first?

- ❍ Yes (1)
- ❍ No (0)

Is there a clear traffic pattern for the grab and go option?

- ❍ Yes (1)
- ❍ No (0)

How would you rate the level of congestion within the area?

	No Congestion (2)	Slightly Congested (1)	Neutral (1)	Congested (0)	Very Congested (0)
Level of Congestion (1)	❍	❍	❍	❍	❍

Are the students greeted by staff?

- ❍ Yes (1)
- ❍ No (0)

Is the staff cheerful?

- ❍ Yes (1)
- ❍ No (0)

Are the students prompted by the staff to take fresh fruit?

- ❍ Yes (1)
- ❍ No (0)

Are the students prompted by the staff to take fresh vegetables?

- ❍ Yes (1)
- ❍ No (0)

Is the serving area clean and free form any spills?

- ❍ Yes (1)
- ❍ No (0)

How would you rate the lighting level?

	No/Low (0)	Low to Medium (1)	Medium (1)	Bright (2)	Very Bright (2)
Lighting Level (1)	❍	❍	❍	❍	❍

How would you rate the noise level?

	No/Low (2)	Low to Medium (1)	Medium (1)	Loud (0)	Very Loud (0)
Noise Level (1)	❍	❍	❍	❍	❍

Is the payment method efficient?

- ❍ Yes (1)
- ❍ No (0)
